# Ocular infection of mice with an avirulent recombinant HSV-1 expressing IL-4 and an attenuated HSV-1 strain generates virulent recombinants in vivo

**Published:** 2010-10-26

**Authors:** Kevin R. Mott, Steven L. Wechsler, Homayon Ghiasi

**Affiliations:** 1 Center for Neurobiology and Vaccine Development, Ophthalmology Research Laboratories, CSMC Burns & Allen Research Institute, Los Angeles, CA; 2 Virology Research, The Gavin S. Herbert Eye Institute, University of California Irvine, Irvine, CA; 3 Department of Microbiology and Molecular Genetics, University of California Irvine, Irvine, CA; 4 The Center for Virus Research, University of California Irvine, Irvine, CA

## Abstract

**Purpose:**

To assess the relative impact of overexpression of interleukin 2 (IL-2), interleukin 4 (IL-4), and interferon gamma (IFN-γ) expressing recombinant herpes simplex virus type 1 (HSV-1) on altering immune responses in ocularly infected mice.

**Methods:**

BALB/c mice were co-infected ocularly with avirulent HSV-1 strain KOS and avirulent recombinant HSV-1 expressing murine IL-4 (HSV-IL-4). Controls mice were co-infected with KOS + HSV-IL-2 or KOS + HSV-IFNγ. Following ocular infection, virus replication in the eye, corneal scarring (CS), and survival were determined. We also isolated recombinant viruses from eye and trigeminal ganglia of KOS + HSV-IL-4 infected mice.

**Results:**

In this study we found that ocular infection of BALB/c mice with a mixture of HSV-IL-4 and KOS resulted in increased death and increased eye disease. In contrast, when mice were infected in one eye with KOS and the other eye with HSV-IL-4 no death or eye disease was seen. Intraperitoneal co-infection of mice with KOS and HSV-IL-4 also did not result in HSV-1 induced death. Interestingly, ocular infection of mice with a mixture of HSV-IL-2 and KOS did not have any effect on severity of the disease in infected mice. We isolated recombinant viruses from KOS + HSV-IL-4 infected mice eye and trigeminal ganglia. Some of the isolated viruses were more neurovirulent then either parental virus. Infection of macrophages with IL-4 expressing virus down-regulated IL-12 production by macrophages.

**Conclusions:**

These results suggest a role for IL-4 in suppression of immune response and generation of virulent viruses in vivo.

## Introduction

Herpes Simplex virus type 1 (HSV-1) is a neurotropic virus that spreads from the site of infection (i.e., eye, genital tract, labial) to the nervous system [1]. In both humans and animal models of HSV-1, virus establishes a latent infection in the ganglia [2]. Based on neurovirulence in animal studies, HSV-1 strains can be classified into two main categories: (1) Avirulent HSV-1 strains, such as strain KOS, do not kill BALB/c mice or New Zealand White (NZW) rabbits following ocular infection; and (2) virulent HSV-1 strains, such as McKrae, that kill ~50% or more BALB/c mice and NZW rabbits following ocular infection [3-6]. Previously it was shown that footpad infection of mice with a 1:1 mixture of avirulent HSV-1 strains ANG and KOS resulted in a lethal infection in 62% of the infected mice [7,8]. The avirulent phenotype in ANG and KOS appeared to be the result of single amino acid changes to glycoprotein D (gD) or gB, respectively [9,10].

In contrast, to *HSV-1* essential genes and the *γ34.5* virulence gene [9-11], deletion of the latency associated transcript (LAT) does not alter virulence despite reducing reactivation in ocularly infected rabbits and mice [12-14]. Using the McKrae derived *LAT*-deficient virus dLAT2903 [12], we previously constructed recombinant viruses expressing murine IL-2 (HSV-IL-2) and IL-4 (HSV-IL-4), each driven by the *LAT* promoter [15,16]. These recombinant viruses, in contrast to their parental virus, were avirulent in ocularly infected mice despite having similar replicating kinetics in tissue culture [15,16]. The HSV-IL-2 recombinant virus, but not the HSV-IL-4 recombinant virus, induced central nervous system (CNS) demyelination following ocular infection of mice [17,18]. In this study we set out to determine if co-infection with KOS or HSV-IL-4 would block HSV-IL-2-induced CNS demyelination. Surprisingly, following ocular infection of BALB/c mice with a mixture of KOS and HSV-IL-4, 43% of the infected mice died. We isolated four viruses from trigeminal ganglia and corneas of mice with severe neurologic involvement. These viruses showed a wide range of virulence and corneal scarring. Virulent recombinant viruses were only generated using ocular co-infection of HSV-IL-4 with KOS, and not KOS with HSV-IL-2, HSV-CD80, HSV-IFNγ, HSV-IL-12p35, or HSV-IL-12p40 recombinant viruses.

## Methods

### Virus, cells, and mice

Plaque-purified HSV-1 strains, KOS, McKrae, dLAT2903 [12], DM33 [19], HSV-IL-4, and dbl-IL-4 [20,21] recombinant viruses were grown in rabbit skin (RS) cell monolayers in minimal essential medium (MEM) containing 5% fetal calf serum (FCS), as described previously [22]. McKrae (wild type parental virus for dLAT2903) and dLAT2903 (*LAT*[-] parental virus for HSV-IL-4 and DM33) viruses are virulent at an infectious dose of 2×10^5^ plaque forming units (PFU)/eye, causing obvious acute eye disease in BALB/c mice and NZW rabbits, and killing ~80% of BALB/c mice and ~50% of NZW rabbits. In contrast, KOS, DM33 (LAT(-) and γ34.5 (-) parental virus for dbl-IL-4, *LAT*(-) HSV-IL-4, and *LAT*(-) and γ34.5 (-)dbl-IL-4 viruses are severely attenuated. All viruses plaque purified 8 times. BALB/cJ (female, 6-week-old) mice were obtained from The Jackson Laboratory (Bar Harbor, ME). Animals were handled in accordance with the ARVO statement for the Use of Animals in Ophthalmic and Vision Research.

### Ocular infection

Mice were infected ocularly with a mixture of 1×10^5^ PFU of KOS plus 1×10^5^ PFU of HSV-IL-4, or dbl-IL-4 per eye in 5 μl of tissue culture media as eye drops without prior corneal scarification. Some mice were infected with 2×10^5^ PFU/ eye of KOS in one eye and 2×10^5^ PFU/ eye of HSV-IL-4 in the other eye. Control mice were infected with 2×10^5^ PFU/ eye of KOS, HSV-IL-4, or dbl-IL-4.

### Evaluation of corneal scarring

Clinical eye disease patterns were determined by examining the eyes of the mice on day 28 post infection. HSV-induced corneal scarring (epithelial keratitis) was evaluated by slit lamp biomicroscopy using 1% fluorescein stain. The magnitude of stromal disease was scored as 0.25, 0.5, 0.75, 1, 1.5, 2, 2.5, 3, 3.5, or 4, with 0, 1, 2, 3, and 4 representing no disease and disease involving 25, 50, 75, and 100% of the corneal surface, respectively.

### Analysis of replication and clearance of HSV-1 from the eye

Eyes were swabbed once daily on days 1, 3, and 5 post-ocular infection with a Dacron swab (Spectrum type 1). The swab was transferred to a 12×75 mm culture tube containing 1 ml of media, frozen, thawed, and virus titers determined using standard plaque assays on RS cells.

### Infection of bone marrow (BM)-derived macrophages in vitro

Monolayers of macrophages isolated from BALB/c mice were infected with 10 PFU/cell of dLAT2903 (HSV-IL-4 parental virus), HSV-IL-4, or mock-infected. One hour after infection at 37 °C, virus was removed and the infected cells were washed three times with fresh media and fresh media was added to each well. The monolayers including the media were harvested at 12 and 24 h post infection. RNA preparation was done as we previously described [23].

### TaqMan Real-Time PCR

The expression levels of IL-12p35 and IL-12p40 genes, along with the expression of the cellular glyceraldehyde-3-phosphate dehydrogenase (*GAPDH*) gene (internal control) were evaluated using commercially available TaqMan Gene Expression Assays (Applied Biosystems, Foster City, CA) with optimized primer and probe concentrations as we previously described [23,24]. Primer-probe sets consisted of two unlabeled PCR primers and the FAM™ dye-labeled TaqMan MGB probe formulated into a single mixture. The primers and probe used were as follows: 1) IL-12p35 (ABI ASSAY I.D. Mm00434165_m1 – Amplicon length=68 bp); 2) IL-12p40 (ABI ASSAY I.D. Mm 01288992_m1 – Amplcon length=109 bp); and 3)IL-4 (ABI Mm00445259_m1 amplicon length=79 bp). *GAPDH* was used as an internal control (ABI ASSAY I.D. m999999.15_G1 - Amplicon Length=107 bp). The expression level of HSV-1 *gB* was similarly evaluated using custom made TaqMan Gene Expression Assays (Applied Biosystems). The *gB* primers and probe were: forward primer, 5′-AAC GCG ACG CAC ATC AAG-3′; reverse primer, 5′-CTG GTA CGC GAT CAG AAA GC-3′; and probe, 5′-FAM-CAG CCG CAG TAC TAC C-3′. Quantitative real-time PCR was performed as we described previously [23]. Real-time PCR was performed in triplicate for each sample from each time point. Relative gene expression levels were normalized to the expression of the *GAPDH* housekeeping gene (endogenous loading control).

### Southern analyses

Briefly, viral DNA was digested with BamHI, the restriction fragments were separated in a 0.9% agarose gel, transferred to Zeta paper, rinsed in 2× SSC (1× SSC is 0.15 M NaCl plus 0.015 M sodium citrate) for 5 min, cross-linked to the membrane by UV light, and DNA-DNA hybridization performed with ^32^P-labeled IL-4 DNA as previously described [15,25].

### Statistical analysis

Fisher’s exact tests were performed using the computer program Instat (GraphPad, San Diego, CA) to analyze survival and corneal scarring (CS). Results were considered statistically significant when the “p” value was <0.05.

## Results

### Co-infection of BALB/c mice with avirulent HSV-IL-4 and KOS increases virulence in infected mice

Groups of 70 BALB/c mice from 7 different experiments were infected ocularly with 2×10^5^ PFU/eye of HSV-IL-4 and KOS at a 1:1 ratio, while 20 control mice per group from 4 separate experiments were infected ocularly with 2×10^5^ PFU/eye of each virus as described in the Methods. All mice (100%) infected with each individual virus (HSV-IL-4 or KOS) survived ocular infection (Table 1). In contrast, only 43% (30/70) mice infected with a mixture of HSV-IL-4 and KOS survived. This difference between mice infected with a mixture of HSV-IL-4 and KOS compared with mice infected with each individual virus was highly significant (p=0.0001, Fisher’s exact test). In contrast to the co-infection results, when mice were infected with KOS in the right eye and HSV-IL-4 in the left eye no increase in virulence was observed in infected mice (not shown). In addition, when mice were co-infected with a mixture of KOS and HSV-IL-2 (instead of KOS and HSV-IL-4) no increase in virulence was detected (not shown).

**Table 1:** Mortality of BALB/c mice following ocular infection with mixture of HSV-1.

**Virus**	**Mortality**	**p-value**
HSV-IL-4+KOS	30/70 (43%)	
HSV-IL-4	0/20 (0%)	0.0001 (HSV-IL-4+KOS versus HSV-IL-4)
KOS	0/20 (0%)	0.0001 (HSV-IL-4+KOS versus HSV-IL-4)
dbl-IL-4+KOS	2/30 (7%)	0.51 (dbl-IL-4+KOS versus dbl-IL-4)
dbl-IL-4	0/20 (0%)	

BALB/c mice were infected ocularly with 2x10^5^ PFU/eye of each virus or a mixture of two viruses. Survival was determined 28 days post infection as described in Materials and Methods. Survival for HSV-IL-4, KOS, dbl-IL-4+KOS, or dbl-IL-4 is from four separate experiments, while the data for HSV-IL-4+KOS is from 7 separate experiments. The p-value was calculated using Fisher exact.

To determine if the increased virulence was associated with IL-4, additional groups of 30 mice (from 4 separate experiments) were co-infected with dbl-IL-4 and KOS. Control mice were infected with dbl-IL-4 alone. One hundred percent of the mice infected with dbl-IL-4 survived the infection at both doses (20/20; Table 2), while 7% (2/30) of mice infected with the dbl-IL-4 + KOS died (Table 2). Although this difference did not reach statistical significance, it should be noted that the dbl-IL-4 parent virus DM33, is deleted for γ34.5 and LAT, and neither this virus, nor d34.5, deleted for γ34.5, nor KOS, has ever killed a single mouse or rabbit in our hands. Thus, the death of 2 mice with the mixture of dbl-IL-4 + KOS may suggest that this virus mixture was more virulent than either parent. However, we cannot rule out that the death of these 2 mice could be due to other reasons as well. We therefore conclude that mixtures of KOS + a virus expressing IL-4 driven by the LAT promoter resulted in decreased survival (i.e., increased virulence).

**Table 2:** Mortality of BALB/c mice following ocular infection with viruses isolated from eye or TG of co-infected mice.

**Virus**	**Mortality**
vEye2	0/20 (0%)
vTG2	0/20 (0%)
vEye3	16/20 (80%)
vTG3	4/20 (20%)

BALB/c mice were infected ocularly with 2×10^5^ PFU/eye of each virus isolated from eye or TG of mice following co-infection with HSV-IL-4+KOS mixtures described in Table 1. Survival was determined 28 days post infection as described in the Methods.

### Virus replication in mouse tears

**Figure 1:**
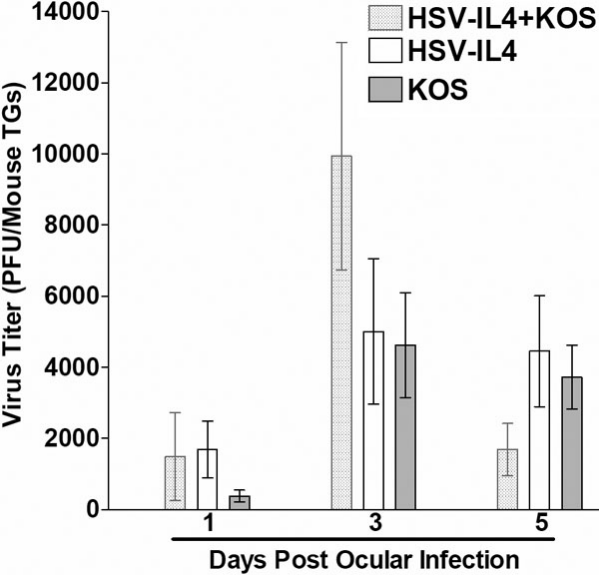
Virus titers in the eyes of mice following ocular infection. Mice were ocularly infected with HSV-IL-4 + KOS, HSV-IL-4, or HSV-1 strain KOS as described in the Methods. The presence of HSV-1 in tear films was monitored on days 1, 3, and 5 post-infection, as described in Methods section. Each data point represents the average virus titer from 40 eyes (y-axis). Data are expressed as average±SEM.

**Figure 2:**
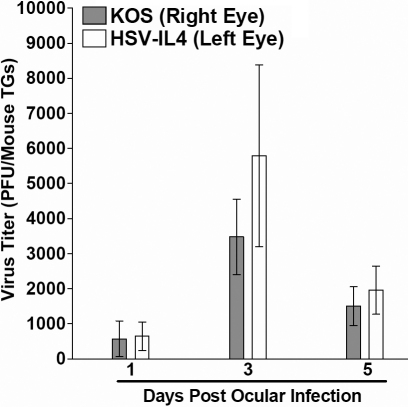
Virus titers in the eyes of mice following ocular infection. Mice were ocularly infected with HSV-1 strain KOS on the right eye and HSV-IL-4 on the left eye. The presence of HSV-1 in tear films was monitored on days 1, 3, and 5 post-infection, as described in Methods section. Each data point represents the average virus titer from 20 eyes (y-axis). Data are expressed as average±SEM.

The virus titers in the tear films that had been collected on days 1, 3, and 5 post ocular infection from mice described in Table 1 were determined using plaque assays on RS cells. There were no significant differences among the virus titers in the tear films of mice infected with HSV-IL-4 + KOS compared with mice infected with KOS alone or HSV-IL-4 alone (Figure 1). Similarly no significant differences were detected in mice that were infected in their right eye with KOS compared with the same mice that were infected with HSV-IL-4 on the left eye (Figure 2). Thus, it appears that there was no direct correlation between acute virus replication in the eye on days 1, 3, or 5 PI and increased virulence in co-infected mice.

### Corneal scarring (CS) in surviving mice

**Figure 3:**
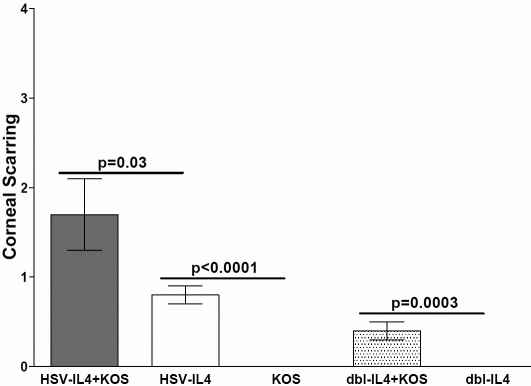
Corneal scarring in co-infected mice. Corneal scarring in surviving mice described in Table 1 was examined on day 30 PI as described in the Methods. CS score represents the average±SEM from 80, 40, 40, 56, and 40 eyes infected with HSV-IL-4+KOS, HSV-IL-4, KOS, dbl-IL-4+KOS, and dbl-IL-4, respectively.

CS was measured in all mice that survived until 28 days after ocular infection (Table 1). The extent of CS was significantly higher in mice co-infected with HSV-IL-4+KOS than mice infected with either HSV-IL-4 or KOS separately (Figure 2; p=0.03 and p<0.0001, respectively). Similarly CS was significantly higher in mice that were co-infected with dbl-IL-4+KOS than mice infected with dbl-IL-4 or KOS separately (Figure 3; p=0.0003). Thus, co-infection of mice with KOS and two different recombinant viruses expressing IL-4, increased severity of CS in surviving mice.

### Virulence and CS with viruses isolated from eyes and trigeminal ganglia of co-infected mice

HSV-1 was isolated from eyes and TGs of mice co-infected with HSV-IL-4 + KOS, following euthanasia on day 6 post infection. Tissues were ground up and total supernatants were grown on RS cells as described in the Methods. Viral supernatants were plaque purified and after three cycles of plaque purification, four of the plaque purified viruses isolated from eyes and TGs were used for further study. Groups of 20 BALB/c mice were infected ocularly with 2×10^5^ PFU/eye of each of the 4 plaque purified viruses (i.e., vEye2, vTG2, vEye3, or vTG3). All mice (100%) infected with vEye2 or vTG2 virus survived ocular infection (Table 2). In contrast, only 80% (16/20) and 20% (4/20) of mice infected with vEye3 and vTG3 survived ocular infection, respectively. This difference between mice infected with vEye3 compared with mice infected with each individual virus was highly significant (p=0.0001, Fisher’s exact test).

**Figure 4:**
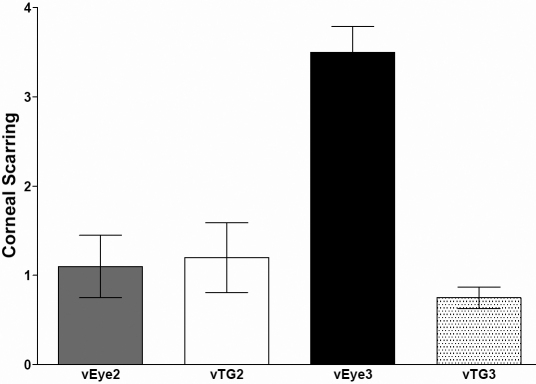
Corneal scarring in mice infected with recombinant viruses. Corneal scarring in surviving mice described in Table 2 was examined on day 30 PI as described in the Methods. CS score represents the average±SEM from 40, 40, 8, and 32 eyes infected with vEY2, vTG2, vEye3, or vTG3, respectively.

CS was measured in surviving mice shown in Table 2. The Level of CS for mice infected with vEy2, vTG2, and vTG3 was the same as mice co-infected with HSV-IL-4+KOS (Figure 4; p>0.05). However, CS in mice that were infected with vEye3 virus was significantly higher than other groups or co-infected mice described in Table 1 (Figure 4; p<0.001). Thus, as a result of co-infection we have isolated a virus that is more pathogenic than either individual parental virus or co-infection with a mixture of both parental viruses.

### Structure of isolated viruses

**Figure 5:**
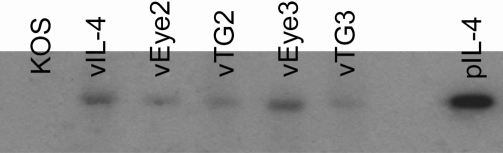
Southern analyses of isolated viruses. Subconfluent RS cell monolayers were infected with 10 PFU/cell of KOS, HSV-IL-4, vEye2, vTG2, vEye3, and vTG3 viruses for 16 h. Viral DNAs were isolated, 5 μg of DNA/each virus was digested with BamHI, and hybridized to ^32^P-labeled murine IL-4. pLAT-IL-4 containing the full-length IL-4 was used as positive control. Lanes: KOS, HSV-IL-4, vEye2, vTG2, vEye3, vTG3, and pLAT-IL-4.

HSV-IL-4 was derived from the dLAT2903 strain by the insertion of the *IL-4* gene and restoration of the *LAT* promoter so that the inserted *IL-4* gene is under control of the endogenous *LAT* promoter [15]. To determine if vEye2, vEye3, vTG2, and vTG3 still contain the *IL-4* insert, the genomic structure of each virus was confirmed by restriction enzyme analysis, and Southern blot (Figure 5). Similar to HSV-IL-4, the vEye2, vEye3, vTG2, and vTG3 viruses all had the *IL-4* insert. The size of the *IL-4* insert was similar to that of *IL-4* from pLAT-IL-4 (Figure 5). As expected KOS DNA was negative for presence of *IL-4* (Figure 5). Thus, the size of the *IL-4* gene in the isolated recombinant viruses was similar to the *IL-4* gene in the parental HSV-IL-4 virus.

**Figure 6:**
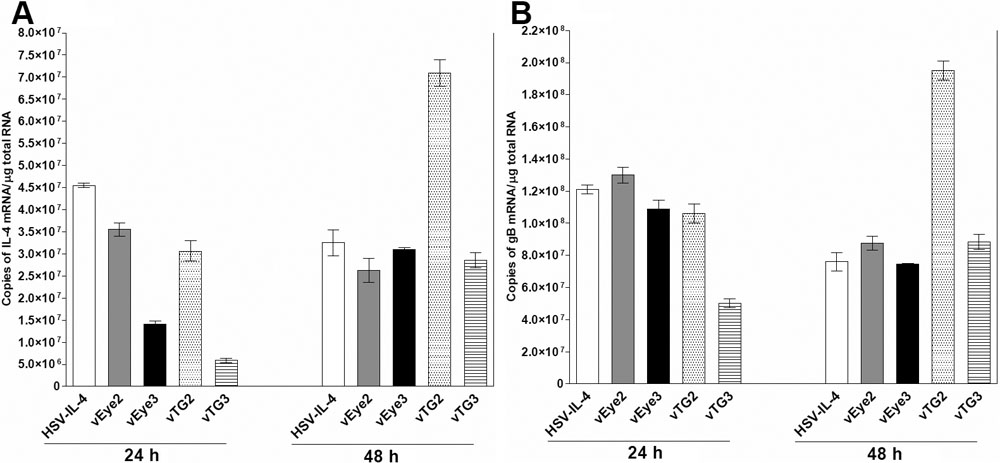
Level of *IL-4* and HSV-1 *gB* transcripts in RS cells infected with different recombinant viruses. Subconfluent monolayers of RS cells were infected with 10 PFU/cell of HSV-IL-4, vEye2, vEye3, vTG2, or vTG3. Total RNA was isolated 24 and 48hr post infection and TaqMan RT–PCR was performed using *IL-4*- and *gB*-specific primers as described in the Methods. In each experiment, an estimated relative copy number of *IL-4* and *gB* were calculated using standard curves generated from pVR1055-IL-4 and pVR1055-gB, respectively. Briefly, DNA template was serially diluted 10-fold such that 5 μl contained from 10^3^ to 10^11^ copies of *IL-4* or *gB*, then subjected to TaqMan PCR with the same set of primers. By comparing the normalized threshold cycle of each sample to the threshold cycle of the standard, the copy number for each reaction was determined. *GAPDH* was used as internal control. Each point represents the mean±SEM (n=4). Panel **A** indicated gB and panel **B** indicates IL-4.

To confirm that the *LAT* promoter was functional in the isolated viruses, confluent monolayers of RS cells were infected at a multiplicity of 10 PFU/cell of HSV-IL-4, vEye2, vEye3, vTG2, or vTG3. Infected cells were collected 24 and 48 h post infection and total RNA was isolated for detection of the *IL-4* transcript by TaqMan RT–PCR as described in the Methods. At 48 h post infection, the levels of *IL-4* transcript were similar for all viruses, except vTG3, which appeared higher (Figure 6A; 48 h). This suggested that the increased neurovirulence of vEye3 was not due to decreased expression of *IL-4* transcript at this time. However, at 24 h post infection the level of *IL-4* transcript was reduced with vEye3 compared to parental HSV-IL-4 (Figure 6A; 24 h). Thus, it is possible, but we think unlikely, that reduced *IL-4* expression early in infection could be involved with increased neurovirulence of vEye3. HSV *gB* transcript levels were examined as a control (Figure 6B). The *gB* RNA levels followed the same patterns seen for *IL-4* RNA, except for vEye2 which had *gB* RNA levels similar to the parental virus at 24 h post infection. Similar patterns of *IL-4* RNA levels were detected when RS cells were infected for 12 h or 24 h with 1PFU/cell of each virus (not shown). To confirm that the *IL-4* transcripts were being translated into protein, the media from the infected RS cells described above were subjected to ELISA as we described previously [20]. All four viruses appeared to express similar levels of IL-4 (not shown). Together, these results suggest that the observed increased virulence detected with the isolated recombinant virus vEYe2 was not due to reduced expression of IL-4 compared to the parental HSV-IL-4 virus.

### Down-regulation of IL-12p35 and IL-12p40 transcripts in BM-derived macrophages infected with HSV-IL-4

**Figure 7:**
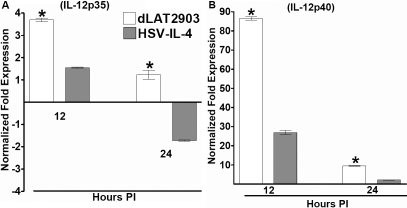
Level of *IL-12p35* and *IL-12p40* transcripts in macrophages infected with HSV-IL-4. Subconfluent monolayers of macrophages were infected with 10 PFU/cell of HSV-IL-4 or parental virus. Total RNA was isolated 12 and 24 h post infection and TaqMan RT–PCR was performed using *IL-12p35*- and *IL-12p40*-specific primers as described in the Methods. *IL-12p35* and *IL-12p40* mRNA levels were normalized in comparison to each transcript in mock-infected cells. *GAPDH* was used as internal control. Each point represents the mean±SEM (n=8).

Since IL-4 is an indicator of T_H_2 response and macrophages play a major role in pushing the immune response toward T_H_1 and away from T_H_2 by IL-12 production, we investigated the possibility of whether HSV-IL-4 suppresses IL-12p35 and IL-12p40 transcripts. Macrophages were isolated from BALB/c mice and infected with 10 PFU/cell of HSV-IL-4, dLAT2903, or mock infected. Infected or mock-infected macrophages were harvested 12 and 24 h post infection and total RNA was isolated as described in Materials and Methods. The levels of *IL-12p35* and *IL-12p40* mRNAs were quantitated by TaqMan RT–PCR. Cellular *GAPDH* mRNA was used as an internal control. Our results suggest that compared to dLAT2903, HSV-IL-4 suppressed expression of both *IL-12–35* (Figure 7A) and *IL-12p40* transcripts (Figure 7B). The pattern of *IL-12p35* and *IL-12p40* transcript in KOS infected macrophages were similar to that of dLAT2903 (not shown). These results suggest that HSV-IL-4 infection suppresses *IL-12* responses in infected macrophages and this may skew the T_H_1 response toward a T_H_2 response.

## Discussion

IL-4 has a broad range of biologic and immunological activities [26,27] and is considered an indicator of a T_H_2 response [27-29]. IL-4 is secreted by activated CD4^+^ T_H_2 cells [30], CD8^+^ T_C_2 cells [31], mast cells [32], and basophils [33,34]. In this study, we have shown that ocular infection of mice with a mixture of two avirulent HSV-1 viruses, in which one of the viruses expresses murine IL-4 increased viral pathogenesis. In contrast, when we co-infected mice with recombinant viruses expressing other cytokine genes and HSV-1 strain KOS no increase of pathogenesis and neurovirulence was detected in infected mice. Our co-infection result is similar to mousepox virus expressing IL-4 which has increased virulence [35]. This may be because the mousepox virus expressing IL-4 resulted in reduced *IFN-γ* gene expression [35].

Although IL-4 enhances T_H_2 development [27,28], however the effect of IL-4 expressed by recombinant HSV-1 on T_H_1 responses may not be a direct effect. Our results suggest that IL-4 has a suppressive effect on IL-12 expression, while previously it was shown that exogenous application of IL-4 is upregulating the production of IL-12 [36]. This discrepancy could be due to use of a recombinant virus expressing IL-4 rather then adding rIL-4 to the culture. Interleukin-12 (IL-12) is a pleiotropic heterodimeric glycoprotein composed of a 35-kDa α subunit and a 40-kDa β subunit [37,38]. The IL-12 heterodimer may bias the response in favor of the production of T_H_1 cells through its ability to drive the differentiation of T_H_0 cells into T_H_1 cells [39-41]. Thus, our results may suggest that IL-4 suppression of IL-12 may bias the T_H_1 response toward a T_H_2 response and this may lead to increase of recombination in vivo. In line with this finding, previously we have shown that HSV-1 replicated to higher titers in the eyes of IL-2^−/−^ mice which have higher T_H_2 response then WT or IL-4^−/−^ mice [42]. Furthermore, we have reported that in IL-4^−/−^ mice, which are deficient in IL-4 production, lack a T_H_2 response, and have elevated IL-2 response, HSV-1 replicated to lower titers and ocular HSV-1 replication could be increased by exogenously added rIL-4 [42]. Previous studies also have shown that delayed viral clearance was seen in mice challenged with influenza virus in the presence of exogenously applied IL-4 [43], following respiratory syncytial virus infection of transgenic mice expressing IL-4 [44], and following infection of mice with a vaccinia virus recombinant expressing IL-4 [45]. Thus, the present study suggests that IL-4 expressed by HSV-1 increases virus recombination by shifting the immune response from a T_H_1 to a T_H_2. Similar to this study, in another study, IL-4 expression by a recombinant vaccinia virus exacerbated infection and the IL-4-induced exacerbation was T cell independent [46].

In summary, co-infection of two avirulent HSV-1 in which one of the two viruses expressing IL-4 generated recombinant viruses in vivo. These recombinant viruses were more pathogenic and more virulent then their parental viruses. Infection of macrophages with IL-4 expressing virus down-regulated IL-12 production by macrophages. These findings suggest a role for IL-4 in suppression of immune response and generation of virulent viruses in vivo.
